# Supplementary material for: Modeling glioblastoma heterogeneity as a dynamic network of cell states
Source: Mol Syst Biol. 2021 Sep 16;17(9):e10105. doi: 10.15252/msb.202010105 (PMC8444284; doi:10.15252/msb.202010105)
Supplement: Supplementary file 6 — Source Data for Figure 5 [file MSB-17-e10105-s004.zip › Figure5A_sourcedata/GSEA_3071/hallmarks_stateII.GseaPreranked.1623742536692/HALLMARK_APICAL_JUNCTION.html]

Details for gene set HALLMARK\_APICAL\_JUNCTION[GSEA]

|  || Dataset | state23071 |
| Phenotype | NoPhenotypeAvailable |
| Upregulated in class | na\_pos |
| GeneSet | HALLMARK\_APICAL\_JUNCTION |
| Enrichment Score (ES) | 0.28906482 |
| Normalized Enrichment Score (NES) | 1.5770736 |
| Nominal p-value | 0.015873017 |
| FDR q-value | 0.05004173 |
| FWER p-Value | 0.401 |
Table: GSEA Results Summary

  

Fig 1: Enrichment plot: HALLMARK\_APICAL\_JUNCTION      
 Profile of the Running ES Score & Positions of GeneSet Members on the Rank Ordered List

  

| PROBE | GENE SYMBOL | GENE\_TITLE | RANK IN GENE LIST | RANK METRIC SCORE | RUNNING ES | CORE ENRICHMENT || 1 | NF2 |  |  | 103 | 1.036 | 0.0042 | Yes |
| 2 | CD274 |  |  | 119 | 1.008 | 0.0293 | Yes |
| 3 | FBN1 |  |  | 143 | 0.972 | 0.0515 | Yes |
| 4 | ACTG2 |  |  | 154 | 0.947 | 0.0762 | Yes |
| 5 | LAYN |  |  | 189 | 0.885 | 0.0931 | Yes |
| 6 | EGFR |  |  | 198 | 0.874 | 0.1162 | Yes |
| 7 | FLNC |  |  | 202 | 0.872 | 0.1405 | Yes |
| 8 | MYL9 |  |  | 247 | 0.827 | 0.1533 | Yes |
| 9 | ACTN1 |  |  | 256 | 0.813 | 0.1746 | Yes |
| 10 | YWHAH |  |  | 273 | 0.781 | 0.1930 | Yes |
| 11 | VCL |  |  | 278 | 0.778 | 0.2143 | Yes |
| 12 | ITGA3 |  |  | 286 | 0.775 | 0.2348 | Yes |
| 13 | MYH9 |  |  | 323 | 0.735 | 0.2470 | Yes |
| 14 | NEXN |  |  | 362 | 0.702 | 0.2577 | Yes |
| 15 | CALB2 |  |  | 485 | 0.636 | 0.2457 | Yes |
| 16 | MAP3K20 |  |  | 587 | 0.583 | 0.2374 | Yes |
| 17 | TUBG1 |  |  | 617 | 0.570 | 0.2465 | Yes |
| 18 | EPB41L2 |  |  | 623 | 0.569 | 0.2616 | Yes |
| 19 | HADH |  |  | 706 | 0.530 | 0.2564 | Yes |
| 20 | TSPAN4 |  |  | 753 | 0.515 | 0.2598 | Yes |
| 21 | ACTG1 |  |  | 755 | 0.514 | 0.2743 | Yes |
| 22 | CAP1 |  |  | 756 | 0.514 | 0.2891 | Yes |
| 23 | CNN2 |  |  | 848 | 0.485 | 0.2804 | No |
| 24 | NEGR1 |  |  | 878 | 0.477 | 0.2869 | No |
| 25 | MYH10 |  |  | 985 | 0.448 | 0.2734 | No |
| 26 | ITGA2 |  |  | 1114 | 0.415 | 0.2536 | No |
| 27 | AMIGO2 |  |  | 1216 | 0.394 | 0.2398 | No |
| 28 | ZYX |  |  | 1399 | 0.358 | 0.2050 | No |
| 29 | CDH11 |  |  | 1440 | 0.349 | 0.2051 | No |
| 30 | PFN1 |  |  | 1488 | 0.342 | 0.2032 | No |
| 31 | HRAS |  |  | 1538 | 0.334 | 0.2006 | No |
| 32 | ACTB |  |  | 1565 | 0.330 | 0.2036 | No |
| 33 | SHC1 |  |  | 1671 | 0.314 | 0.1866 | No |
| 34 | IKBKG |  |  | 1731 | 0.307 | 0.1808 | No |
| 35 | VCAN |  |  | 1793 | 0.299 | 0.1742 | No |
| 36 | ARPC2 |  |  | 1800 | 0.299 | 0.1813 | No |
| 37 | SLIT2 |  |  | 1873 | 0.289 | 0.1717 | No |
| 38 | SDC3 |  |  | 1908 | 0.284 | 0.1715 | No |
| 39 | VASP |  |  | 1912 | 0.284 | 0.1788 | No |
| 40 | MYL12B |  |  | 2035 | 0.269 | 0.1563 | No |
| 41 | GNAI1 |  |  | 2254 | -0.254 | 0.1095 | No |
| 42 | PKD1 |  |  | 2263 | -0.255 | 0.1149 | No |
| 43 | STX4 |  |  | 2374 | -0.267 | 0.0953 | No |
| 44 | EXOC4 |  |  | 2431 | -0.275 | 0.0892 | No |
| 45 | FSCN1 |  |  | 2432 | -0.275 | 0.0971 | No |
| 46 | SKAP2 |  |  | 2581 | -0.298 | 0.0690 | No |
| 47 | KCNH2 |  |  | 2610 | -0.302 | 0.0707 | No |
| 48 | MMP2 |  |  | 2762 | -0.328 | 0.0427 | No |
| 49 | SHROOM2 |  |  | 2799 | -0.335 | 0.0433 | No |
| 50 | SIRPA |  |  | 2823 | -0.340 | 0.0474 | No |
| 51 | EVL |  |  | 2927 | -0.360 | 0.0322 | No |
| 52 | PTEN |  |  | 2929 | -0.361 | 0.0423 | No |
| 53 | JAM3 |  |  | 3016 | -0.378 | 0.0318 | No |
| 54 | ADAM23 |  |  | 3033 | -0.380 | 0.0387 | No |
| 55 | CD276 |  |  | 3050 | -0.385 | 0.0458 | No |
| 56 | GAMT |  |  | 3145 | -0.413 | 0.0343 | No |
| 57 | PIK3R3 |  |  | 3151 | -0.414 | 0.0450 | No |
| 58 | CTNNA1 |  |  | 3163 | -0.418 | 0.0542 | No |
| 59 | CDH4 |  |  | 3164 | -0.418 | 0.0662 | No |
| 60 | CADM3 |  |  | 3292 | -0.455 | 0.0477 | No |
| 61 | LAMB3 |  |  | 3384 | -0.491 | 0.0393 | No |
| 62 | ARHGEF6 |  |  | 3530 | -0.560 | 0.0194 | No |
| 63 | MDK |  |  | 3706 | -0.691 | -0.0042 | No |
| 64 | GNAI2 |  |  | 3845 | -0.835 | -0.0145 | No |
| 65 | NRXN2 |  |  | 3907 | -0.961 | -0.0021 | No |
| 66 | LIMA1 |  |  | 4056 | -1.716 | 0.0104 | No |
Table: GSEA details [plain text format]

  

Fig 2: HALLMARK\_APICAL\_JUNCTION: Random ES distribution      
 Gene set null distribution of ES for **HALLMARK\_APICAL\_JUNCTION**
